# Localization of Axonal Motor Molecules Machinery in Neurodegenerative Disorders

**DOI:** 10.3390/ijms13045195

**Published:** 2012-04-24

**Authors:** Fulvio Florenzano

**Affiliations:** Confocal Microscopy Unit, EBRI and S. Lucia Foundation, Via Fosso del Fiorano, 64/65-00143 Rome, Italy; E-Mail: florenza@hsantalucia.it or f.florenzano@ebri.it

**Keywords:** atrophy, axotomy, axonopathy, brain injury

## Abstract

Axonal transport and neuronal survival depend critically on active transport and axon integrity both for supplying materials and communication to different domains of the cell body. All these actions are executed through cytoskeleton, transport and regulatory elements that appear to be disrupted in neurodegenerative diseases. Motor-driven transport both supplies and clears distal cellular portions with proteins and organelles. This transport is especially relevant in projection and motor neurons, which have long axons to reach the farthest nerve endings. Thus, any disturbance of axonal transport may have severe consequences for neuronal function and survival. A growing body of literature indicates the presence of alterations to the motor molecules machinery, not only in expression levels and phosphorylation, but also in their subcellular distribution within populations of neurons, which are selectively affected in the course of neurodegenerative diseases. The implications of this altered subcellular localization and how this affects axon survival and neuronal death still remain poorly understood, although several hypotheses have been suggested. Furthermore, cytoskeleton and transport element localization can be selectively disrupted in some disorders suggesting that specific loss of the axonal functionality could be a primary hallmark of the disorder. This can lead to axon degeneration and neuronal death either directly, through the functional absence of essential axonal proteins, or indirectly, through failures in communication among different cellular domains. This review compares the localization of cytoskeleton and transport elements in some neurodegenerative disorders to ask what aspects may be essential for axon survival and neuronal death.

## 1. Introduction

Axon degeneration is perhaps the most common and dangerous damage after brain injury for several reasons. The white matter constitutes the major portion of the brain volume. All the brain anatomical subdivisions are permeated by axons coming or directed to other brain regions. Axons are very sensitive to damage. Generally, the majority of brain insults are initially restricted only to a brain region. Following the initial damage, the axons passing through or terminating on the damaged region begin to degenerate, propagating the damage to other brain regions. This subsequent damage spreading is often observed to produce clinical complications, which may represent the disease degenerative course and gradually worsen the patient’s quality of life. Further, damage spreading may represent the pathogenic convergence of different types of insults in the same anatomical area on a common neurodegenerative disease mechanism. Finally, damage spreading is a mechanism playing a variable role in probably all neurodegenerative conditions.

## 2. Axon: The Most Sensitive Neuronal Domain

Cells are dependent on active transport to perform physiological processes and maintain vitality. This is particularly true for neuronal cells, which are endowed by long and specialized processes: axons and dendrites. In particular, axons are long and thin structures presenting specialized signaling endings that make contact with spines, and require continuous supply and clearance of materials [1,2]. The structural and functional features of the axon are determined and maintained by the specific positioning of its organelles and proteins, which must be tightly regulated. The different classes of organelles transported within axons behave differently [3], and the signals that control organelle traffic are diverse. As an additional level of complexity, active transport is very often intimately associated with protein and organelles processing and maturation. Thus, the functional architecture of neurons makes them particularly dependent on intracellular transport processes.

**Figure 1:**
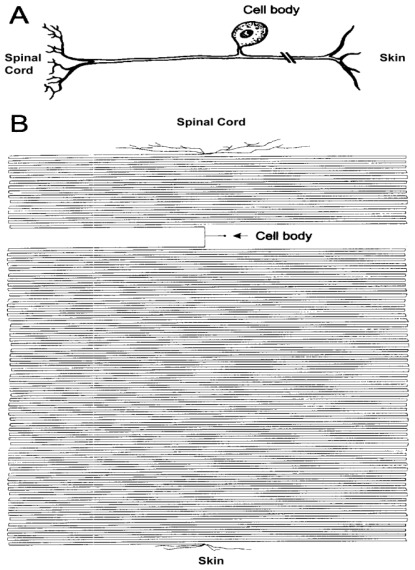
Sketches of a dorsal root ganglion (DRG) neuron. (**A**) Typical textbook representation of a pseudounipolar DRG neuron; (**B**) Proportional drawing of the different cellular regions giving the perception of the geometrical relations among the cell body and the axonal domain. From Devor, M. Unexplained peculiarities of the dorsal root ganglion. *Pain* 1999, *Suppl 6*, S27–S35.

It is generally believed that about 0.2% of the neuronal volume is in the cytoplasmic soma and 99.8% is represented by the axon and dendrites [3,4] (Figure 1). Maintaining such enormous cellular outgrowth and regulating its traffic are probably the major challenges for the nervous system, which is accomplished through the combined support of physiological processes derived from neuronal cell bodies and axon-associated glial cells [5]. The majority of neural tissue volume appears to be composed of axons and dendrites, and these are dependent on active transport from cell bodies to maintain the vitality. This is reflected in aging and in diseases phenomena. In healthy aging, in humans, the major reduction of brain volume is thought to be in the white matter, which contain the axons of projection neurons [6]. Analyses of tissue loss in neurologically normal individuals demonstrate that atrophy is largely confined to the white matter and accounts for about 50% [6], while the total number of cortical neurons in persons ranging from 20 to 90 years varies less than 10% [7].

The other cellular compartment that is severely affected in aging and during neurodegenerative diseases is the dendrite. Loss of spines and consequent dendrite retraction is a common distinguishing mark in several neurodegenerative diseases [8]. Nonetheless, in contrast to axonopathy, a clear “dendritopathy” has still not been characterized; and it is generally believed that damage to a dendritic domain can remain localized without spreading to the other dendritic branches or without generating a retrograde death reaction. The reasons for this resistance can be found in the different dendrite spatial arrangement, but it is highly conceivable that differences in protein transport mechanisms make the dendrites less susceptible to retrograde death phenomena compared to axons. These two cellular domains, axons and dendrites, appear to be the primary sites of damage in aging and after brain injuries.

Aging can be considered a physiological degenerative process while traumatic/vascular injuries can occur frequently during a lifetime. Aging and traumatic/vascular injuries are factors which very often create a general predisposition to degenerative insults and may represent the degenerative frames where insults of different nature trigger pathological reactions and develop their own course. Traumatic/vascular injuries are acute forms of damage that, due to the high percentage of axonal volume in the nervous system, and independently from the specific type of injury, invariably provoke axonal damage. After axonal damage, axons undergo Wallerian degeneration [9,10] or dye back degeneration [9], which ultimately lead to cell bodies atrophy and loss [11]. In the last years the availability of YFP fluorescent mice and *in vivo* imaging techniques allowed direct observations to be made on the early phases after axonal damage. Wallerian degeneration is characterized by focal axon swellings forming varicosities, or larger spheroids, within 6 h of axotomy *in vivo* which extends in a rapid, asynchronous and progressive fashion [12]. Varicosities are arranged in a “beads-on-a-string” pattern resembling the connected, alternating regions of axonal dilation and constriction which have been reported in a wide range of brain diseases [12,13]. For more details on axotomy degeneration processes in wt and (WldS) mutant mice, see [12,13]. These varicosities contain organelles and cytoskeletal proteins suggesting that 6 h after damage axonal transport is disrupted. However, these varicosities did not contain a large accumulation of mitochondria that would have been expected if focal dilation of the axon was caused by impaired mitochondrial transport. This could suggest that early axonal transport deficits are not directly causing swelling [13]. Of note, one of the earliest events after axonal damage is the accumulation of both APP and Abeta proximal to the disrupted segment [14,15]. This occurs before or simultaneously to the appearance of morphological evidence of axonal damage, and in a wide range of disorders [14,15]. The immunoreaction in damaged axons after head trauma remains positive for up to 1 month and becomes positive 1–3 h after the insult [16], suggesting that the impairment of axonal transport and APP processing is one of the earliest events after axonal damage [9,17].

It is difficult to obtain information after the early phase of axonal damage by *in vivo* observations, and can be gathered only by *ex vivo* indirect examinations. After axotomy, two phases of neuronal loss can be described: one confined to the first week and dominated by a high neuronal loss, and a second characterized by a lower neuronal loss and the induction of reactive neuronal and glial phenomena [18–20]. In the second phase, neuronal protein inductions are distributed to the axonal domain demonstrating that protein synthesis and axonal transport are active [18]. Although, after two or three months, neuronal loss is almost complete demonstrating that the reactive neurons are committed to death [21]. Collectively, observations on axotomy experimental models suggest that axonal transport is disrupted when neuronal loss is rapid and passes through the disruption of morphological axonal features. However, when neuronal loss is slow, and not accompanied by evident morphological alterations axonal transport appears to be preserved, although transport impairment cannot be excluded.

## 3. Transport Machinery: Roads, Vehicles and Traffic Regulation

Active transport is executed through cytoskeleton, transport and regulatory elements and the spatial and temporal regulation of motor-based transport is essential to ensure precise cargo delivery. We have gained sufficient knowledge on the structural elements that allow active transport, but how transport is regulated is less well understood. Regulation of the transport traffic can be executed through different mechanisms: comprising posttranslational modifications and phosphorylation of cytoskeleton and transport elements. Besides these molecular modifications, which appear to shape the transport in axonal subdomains, there are specific families of molecules that act in the selection of cargoes.

Cytoskeleton structures are composed of polymers of two different classes of molecules: tubulin and actin. Tubulin polymers are hollow cylinders about 24 nm in diameter (lumen = approximately 15 nm in diameter), most commonly comprising 13 protofilaments which, in turn, are polymers of alpha and beta tubulin [22,23]. These cytoskeletal filaments are formed from the head-to-tail assembly of α- and β-tubulin dimers that serve as tracks for the motors [23]. A characteristic property of these polymeric structures is their ability to undergo cycles of rapid growth and disassembly. This is known as dynamic instability and individual microtubules do not reach a steady-state length, but exist in either polymerization (growth) or depolymerization (shrinkage) states [22]. Tubulin polymers are oriented in a polarized array in the axon, with their plus, or fast growing ends, directed toward axonal endings, and their minus, or slow growing ends, directed toward the cell body. Therefore, anterograde axonal transport is necessarily plus-end directed, whereas retrograde axonal transport is minus-end directed. Polymeric tubulin organization is more complex in dendrites, where the mixed polarity seen in mammalian neurons may contribute to axonal-dendritic sorting and specification [22]. Polymeric tubulin presents two intrinsic mechanisms of regulation: the incorporation of alternative tubulin isoforms and post-translational modification of the tubulin subunits [24]. The other cytoskeletal structures are linear polymers of actin subunits. They are the thinnest filaments of the cytoskeleton acting as tracks for the movement of myosin molecules that attach to the microfilament and “walk” along them [23].

Transport elements are responsible for the intracellular transport of a wide variety of components and for positioning them along the axon with high spatial-temporal precision. Three different classes of motors are involved in this task: dynein and kinesin, which transport cargoes toward the minus and plus ends of microtubules, respectively, and myosin, responsible for the transport along actin filaments. Kinesins and dyneins, which attach on microtubule roads, are an extended superfamily of proteins [25– 27]. Kinesin tail domains are divergent, which allows coupling to a diverse array of cargos. Many kinesins are microtubule-based motors, some are microtubule depolymerizers, while the function of others has yet to be explored. Kinesin proteins (KIFs) comprise several major groups depending on the position of the motor domain within the molecule [28]. Kinesins are known to drive anterograde axonal transport [2] including the three major families kinesin-1 (aka KHC or KIF5), kinesin-2 (aka KIF3), and kinesin-3 (aka KIF1). Kinesins functions are mainly regulated through the phosphorylation state. Two mechanisms for the phosphorylation dependent regulation of kinesins can be indicated. First, kinesin phosphorylation may control the association and dissociation of motors with their cargos. Second, kinesin phosphorylation may modulate the binding of kinesins to microtubules [25,26]. Kinesins often recognize scaffold proteins or adaptor proteins and bind to cargo membrane proteins indirectly as part of a protein complex. In other cases, kinesins directly bind to membrane proteins in the cargo. Three different mechanisms have been implicated in the regulation of the unloading of cargos from kinesins: phosphorylation of kinesins or cargos, control by small G proteins and Ca^2+^ signaling [25,28]. Cytoplasmic dynein is the major minus end-directed microtubule motor in the neuron and is involved in retrograde axonal transport [1]. Dynein is a large protein complex with two heavy chains that form the two motor domains, as well as associated intermediate, light intermediate, and light chains that are involved in cargo recognition and binding specificity.

Regulatory elements are a growing family of molecules, and the list is rapidly expanding [26]. Dynactin is a large, multi-subunit complex required as an activator for most dynein functions in the cell, including retrograde axonal transport. The largest subunit of dynactin is p150Glued, which binds directly to dynein and the microtubule. Multiple mutations in the *DCTN1* gene encoding p150Glued cause neurodegeneration. Vesicular cargos are transported along the axon by both kinesin and dynein motors. The activity of these cargo-bound motors may be co-regulated by scaffolding proteins such as JIPs and Htt/HAP1 that can interact with either kinesin or dynein motors, or potentially with both [26].

An important regulatory point is the management of cargo delivery: which implies the recognition of both the type of cargo and of the specific delivery location. The need to deliver selected materials to specialized axonal subdomains implies the existence of targeting and docking mechanisms. For example, neurotransmitter-bearing synaptic vesicles need to be delivered to presynaptic terminals, whereas vesicles bearing sodium channels should be selectively delivered to nodes of Ranvier [29]. To accomplish this work, neurons rely heavily on phosphorylation-dependent intracellular signaling mechanisms, which help in the spatial and temporal coordination and regulation of many cellular processes within axons, including fast axonal transport [2]. Cumulative data indicates that phosphorylation of molecular motors represents a major mechanism for regulation of fast axonal transport *in vivo* [30,31]. These regulatory mechanisms provide an explanation for delivery of selected motor cargoes to specialized axonal subcompartments. Of particular interest, the activity of many kinases that regulate fast axonal transport are increased in degeneration diseases, as reflected by the aberrant patterns of protein phosphorylation [32]. One of the more striking examples is the increase in Tau phosporylation in several neurodegenerative pathologies [33]. However, the mechanism by which Tau is abnormally phosphorylated and how this is associated with Tau aggregation and intraneuronal accumulation are still not clear. Further, retrograde axonal transport can be altered at multiple levels. Impairment of transport can arise from direct mutations in microtubule motors or their activators and adaptors. Finally, pathological changes along the axon, such as remodeling of the cellular cytoskeleton as an injury adaptation mechanism or the development of protein aggregates, could deleteriously affect retrograde axonal transport.

**Figure 2:**
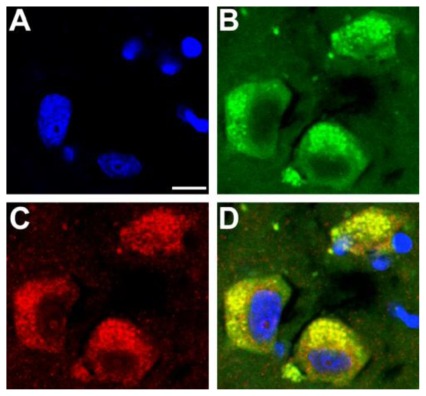
NH2-tau fragment immunoreactivity (green channel) colocalizes with the adenine nucleotide translocator-1 (ANT-1) protein (red channel) in cyoplasmatic mitochondria in Alzheimer’s disease tissue. Note the tau accumulation in fragmented mitochondria surrounding the neuronal nuclei (blue channel). Scale bar 10 μm.

Recently, some papers have linked aberrant protein phosporylation with the disruption of mitochondrial transport and neuronal death in several neurodegenerative diseases. Mitochondria use motors of the kinesin families, along with cytoplasmic dynein, to translocate along microtubules [34]. The decline in mitochondrial functions plays a key role in the aging process and increases the incidence of age-related disorders [35]. Mitochondrial dysfunctions present direct effects on ATP generation, calcium homeostasis, production of reactive oxygen species, and interaction with cytoskeleton proteins [36]. Moreover, Aβ and tau proteins trigger mitochondrial dysfunction through a number of pathways, such as impairment of oxidative phosphorylation, elevation of reactive oxygen species production, alteration of mitochondrial dynamics (Figure 2), and interaction with mitochondrial proteins [37–39].

## 4. Cytoskeleton Composition of Axons Is Related to Environmental Stimuli

Cytoskeleton composition is typical of the specific cellular domains, is primarily related to the fiber caliber, and appears to be shaped by cellular functions and environmental conditions. Spines are primarily made of filamentous actin; dendrites and axons are made up of tubulin polymers. However, their specific compositions are shaped not only by the different functions in relation to the different subcellular domains: but also by the different cellular stimuli eliciting different cellular responses.

The microtubular density (microtubules/μm^2^) of axons is an inverse function of their caliber, and independent of the myelin sheath [40,41]. For a given axonal size, the density is the same irrespective of the length of the axon, the nerve examined, the age of the animal, the developing or regenerating condition of the nerve, the nutritional status, or the species surveyed. Motor and sensory axons span from the cord through the root to the peripheral nerve. For axons of equal size, the microtubular density in the root is half that of the intracord or peripheral domains. This holds for axons regardless of whether they are sensory or motor, myelinated or unmyelinated, spinal or cranial [40–42]. Therefore, axoplasmic proteins are arrayed differently in the root as compared to intracord or peripheral trajectories, *i.e.*, the axonal phenotype correlates with the environment, while the neuronal class (motor, sensitive, or sympathetic) is irrelevant [43]. To investigate the mechanisms of local regulation of the axonal phenotype, axonal central branches were allowed to regenerate along the hypoglossal nerve by means of a surgical anastomosis or, as a control, along its anatomical trajectory. When the central branches regenerated along their anatomical trajectory, the original low microtubular density was found, while upon regeneration along the foreign peripheral nerve, a high density was found. Therefore, the phenotype of the axon correlated with the environment, not with the central nature of the branch [43]. The microtubules and size of axons also are affected by electrical activity. A high and prolonged discharge increases both microtubular content and caliber of axons [41,44]. In conclusion, the molecular composition of the axoplasm varies in relation to its history and its environment.

## 5. Adult Onset Neurodegenerative Diseases Involve Axonopathy and Transport Deficit

Adult onset neurodegenerative diseases are a heterogeneous group of diseases presenting some unifying themes. They are progressive and their clinical phenotype results from an age-dependent decline in selected neuronal populations that is initially associated with loss of synaptic activity and axonopathy and later with neuronal death [2]. They present a common histological picture which is characterized by three main aspects: lesions related to accumulation of proteic products, lesions related to neuronal losses, and reactive processes such as inflammation and plasticity [45]. For most neurodegenerative conditions, injury may originate either from unknown stressors in the case of sporadic disease, or from expression of a mutant gene in the familial forms. Familial and sporadic forms often present nearly indistinguishable clinical course, suggesting pathogenic convergence on common disease mechanisms [2,46]. In addition, all of the genes whose mutation causes the inherited forms of these diseases are widely or ubiquitously expressed in several tissues or in all neurons [2], although their mutation induces degeneration only in a selected neuronal population. The accumulation of organelles and proteins in the cell body and axon are suggestive of axonopathies for many human neurodegenerative diseases. Aggregates of mutant Huntingtin protein (htt) are detected in Huntington’s disease (HD), accumulation of tau and β-amyloid proteins is detected in Alzheimer’s disease (AD), α-synuclein positive Lewy bodies are observed in Parkinson’s disease (PD) and neurofilament accumulations are seen in amyotrophic lateral sclerosis (ALS). These pathologies suggest that impaired axonal transport may underlie the pathogenic accumulations of proteins in neurodegenerative diseases [47].

Clinical deficits in neurodegenerative disorders are associated with the location, rather than mechanism, of brain cell death. The regional selectivity and variability of neuronal loss in different neurodegenerative disorders is reflected in the variety of different and overlapping clinical dementia and movement disorder syndromes characterized by neurodegeneration. While the type and location of cells degenerating in each disorder varies widely, it appears that the range of mechanisms by which the cells die are less broad, with many disorders sharing a small number of neurodegenerative mechanisms in common. Among these the most ubiquitous are protein aggregation, oxidative stress, neuroinflammation and apoptosis [48].

Axonal and cell body accumulation of proteins and organelles are hallmark pathologies for many human neurodegenerative diseases. Tau and β-amyloid are present in several and different types of tissue lesions in Alzheimer’s and related diseases. α-Synuclein is the principal component of Lewy bodies in Parkinson’s disease. Neurofilament accumulation is seen in ALS, and more recently, TDP-43 accumulation has been observed in ALS and frontotemporal lobar degeneration [49–51]. Furthermore, axonal swellings and spheroids have been described in a number of neurodegenerative diseases [9]. Together, such pathologies suggest that defective functioning of the axon contributes to disease and, in particular, that damage to axonal transport may underlie the pathogenic accumulation of organelles. Early axonal and synaptic degeneration in neurodegenerative diseases are consistent with active transport alterations. Evidence indicating that alterations in active transport suffice to produce selective neuronal degeneration comes from genetic studies. Specifically, mutations in genes coding for microtubule-based molecular motor subunits can result in “dying back” neuropathologies [32,52]. Often the length of axons is assumed to correlate with increased vulnerability to active transport defects. Although it is conceivable that vulnerability arises from the length, size and nature of anatomical connections, mutations affecting different subunits of the same motor protein may induce dramatically different pathologies, suggesting additional levels of molecular and cellular complexity [53,54]. Of note, most adult onset neurodegenerative diseases are not associated with mutations in molecular motors, or other proteins, so other mechanisms must be responsible for changes in altered transport [2].

## 6. Conclusions

Neurodegenerative diseases display complex and selective brain topographical distribution of the cellular damage, due to damage spreading following axonal degeneration. The axon is the most sensitive cellular domain and this sensitivity relates to the peculiar spatial arrangement and the high dependence of intracellular transport processes. Transport processes are compromised under neurodegenerative conditions and their restoration may provide therapeutic tools.
